# Transplant-Associated Thrombotic Microangiopathy After Autologous Hematopoietic Stem Cell Transplantation Treated With Eculizumab

**DOI:** 10.7759/cureus.88785

**Published:** 2025-07-25

**Authors:** Taichiro Tokura, Youhei Imai, Reina Saga, Hiroko Hidai, Sayuri Motomura

**Affiliations:** 1 Hematology, Tokyo Metropolitan Tama-Hokubu Medical Center, Tokyo, JPN; 2 Hematology, Nippon Medical School, Tokyo, JPN

**Keywords:** allogeneic hematopoietic stem cell transplantation, autologous hematopoietic stem cell transplantation, cardiopulmonary complications, complement-targeting agents, eculizumab, graft-versus-host disease (gvhd), malignant lymphoma, pulmonary hypertension, right-sided heart failure, transplant-associated thrombotic microangiopathy

## Abstract

Transplant-associated thrombotic microangiopathy (TA-TMA) generally occurs after allogeneic hematopoietic stem cell transplantation (HSCT) and has a high mortality rate. However, TA-TMA after autologous HSCT is rare. In particular, there are almost no accurate reports of TA-TMA after autologous HSCT in adults. Furthermore, there are no reports of such patients being treated with complement-targeting agents, including eculizumab. We report the first adult case of TA-TMA after autologous HSCT treated with eculizumab. A 66-year-old woman in complete remission after the second relapse of follicular lymphoma received autologous HSCT following the MEAM regimen (ranimustine [MCNU], etoposide, cytarabine [Ara-C], and melphalan). Trilineage engraftment was confirmed. However, she developed new-onset hypertension, hemolytic anemia, thrombocytopenia, and renal failure. The screening results were notable for normal coagulation, normal ADAMTS13 activity and inhibitor levels, and negative direct and indirect Coombs tests. No variants appeared in complement regulatory genes such as *CFH*, *CFB*, *CFHR5*, *CFI*, *CD46*, *C3*, *THBD*, and *DGKE*. In addition, all other microbiological and autoimmune screening tests were negative. Based on comprehensive diagnostic findings and the onset within 100 days post-HSCT, TA-TMA was diagnosed. She also developed acute respiratory failure requiring >15 L/min of oxygen. Transthoracic echocardiography revealed pulmonary hypertension (PH) and right-sided heart failure (RHF) as complications of TA-TMA. Following the administration of eculizumab, clinical improvement was observed. In conclusion, TA-TMA after first-time autologous HSCT in adults is extremely rare but can occur. Even with serious complications such as PH and RHF, TA-TMA after autologous HSCT may improve with early diagnosis and prompt initiation of complement-targeting agents, such as eculizumab. These findings suggest that complement inhibition may play a critical therapeutic role even in patients with cardiopulmonary complications and raise the possibility that the prognosis of TA-TMA after autologous HSCT may be more favorable than previously reported in allogeneic HSCT, with a lower mortality rate.

## Introduction

Transplant-associated thrombotic microangiopathy (TA-TMA) typically occurs after allogeneic hematopoietic stem cell transplantation (HSCT), with a mortality rate of 30-50% [1]. The underlying pathophysiology is characterized by vascular endothelial damage leading to systemic complement activation, which exacerbates systemic vascular injury, such as vasculopathy and microvascular thrombosis [2]. Clinically, it causes microangiopathic hemolytic anemia, thrombocytopenia, and microthrombi in the kidneys and lungs, progressing to multiple organ dysfunction syndrome [1,2]. Identified risk factors for vascular endothelial damage include prior HSCT, mismatched donors, acute graft-versus-host disease (GVHD), and myeloablative conditioning, which is intensive chemotherapy and/or total body irradiation (TBI) used to eradicate the patient’s bone marrow before transplantation [2,3]. Accordingly, TA-TMA generally occurs after allogeneic HSCT, which involves a donor, with a pooled incidence of approximately 12% in adult and pediatric recipients [3].

In contrast, TA-TMA is rarely observed after autologous HSCT. This is because less intensive conditioning regimens and the use of autologous cells, which do not lead to GVHD, contribute to less severe vascular endothelial damage. Due to the low incidence of this reason, the exact incidence rate of TA-TMA after autologous HSCT has not been reported. However, in pediatric patients with high-risk neuroblastoma undergoing tandem autologous HSCT, which involves two successive administrations of high-dose intensive chemotherapy and irradiation, TA-TMA is considered more likely to occur because the risk of vascular endothelial injury is elevated [4,5]. Recent studies have also suggested that predisposing conditions, such as variants in complement regulatory genes, including *CFH*, *CFB*, *CFHR5*, *CFI*, *CD46*, *C3*, and *THBD*, may increase susceptibility to TA-TMA, and ongoing research in this area is expected to provide further insights [6]. The diagnostic criteria for TA-TMA were established following consensus by the international expert panel in 2022 [7]. As treatments, withdrawal of drugs potentially causing vascular endothelial damage (such as calcineurin inhibitors and mTOR inhibitors), plasma exchange, rituximab, and defibrotide have been reported, but their effectiveness has been limited [8]. More recently, complement-targeting agents tailored to the pathophysiology of TA-TMA, such as eculizumab, ravulizumab, narsoplimab, pegcetacoplan, and nomacopan, have demonstrated promising efficacy, and several clinical trials are currently underway [1,2,8,9]. In particular, TA-TMA after autologous HSCT does not involve the use of calcineurin inhibitors, which are used to treat GVHD after allogeneic HSCT but may also increase the risk of TA-TMA, thereby potentially simplifying treatment. The prognosis of TA-TMA is quite poor, but almost all reports have referred to TA-TMA after allogeneic HSCT, and the prognosis of TA-TMA after autologous HSCT has not yet been reported.

Here, we report a rare case of TA-TMA that developed in an adult with follicular lymphoma undergoing first-time autologous HSCT without identifiable variants in complement regulatory genes. The patient also developed life-threatening pulmonary hypertension (PH) and right-sided heart failure (RHF) as complications of TA-TMA but showed clinical improvement following eculizumab therapy, which is the first case report of TA-TMA after autologous HSCT in an adult successfully treated with eculizumab.

## Case presentation

A 66-year-old woman had achieved complete remission after the second relapse of follicular lymphoma. Her past medical history was notable for an appendectomy for appendicitis and a total colectomy for ischemic colitis. She had no other significant medical history. All pre-transplant evaluations, including cardiovascular, pulmonary, renal, hepatic, hematologic, endocrine, metabolic, infectious, and autoimmune assessments, were within normal limits.

She received first-time autologous HSCT on October 9, 202X (day 0). The conditioning regimen consisted of MEAM as follows: ranimustine (MCNU) 300 mg/m² (415 mg/body) on day -7; etoposide 200 mg/m² (275 mg/body) from day -6 to -3; cytarabine (Ara-C) 200 mg/m² (275 mg/body) twice daily from day -6 to -3; and melphalan 140 mg/m² (190 mg/body) on day -2. A total of 2.76×10^6^ CD34+ cells/kg were infused on day 0. Trilineage engraftment was confirmed by day 27. The laboratory values at the time of discharge (day 41) were as follows: hemoglobin 8.7 g/dL, platelets 56,000/µL, LDH 162 U/L, creatinine 0.63 mg/dL, and NT-proBNP 134 pg/mL. She was subsequently followed up in the outpatient clinic.

**Figure 1:**
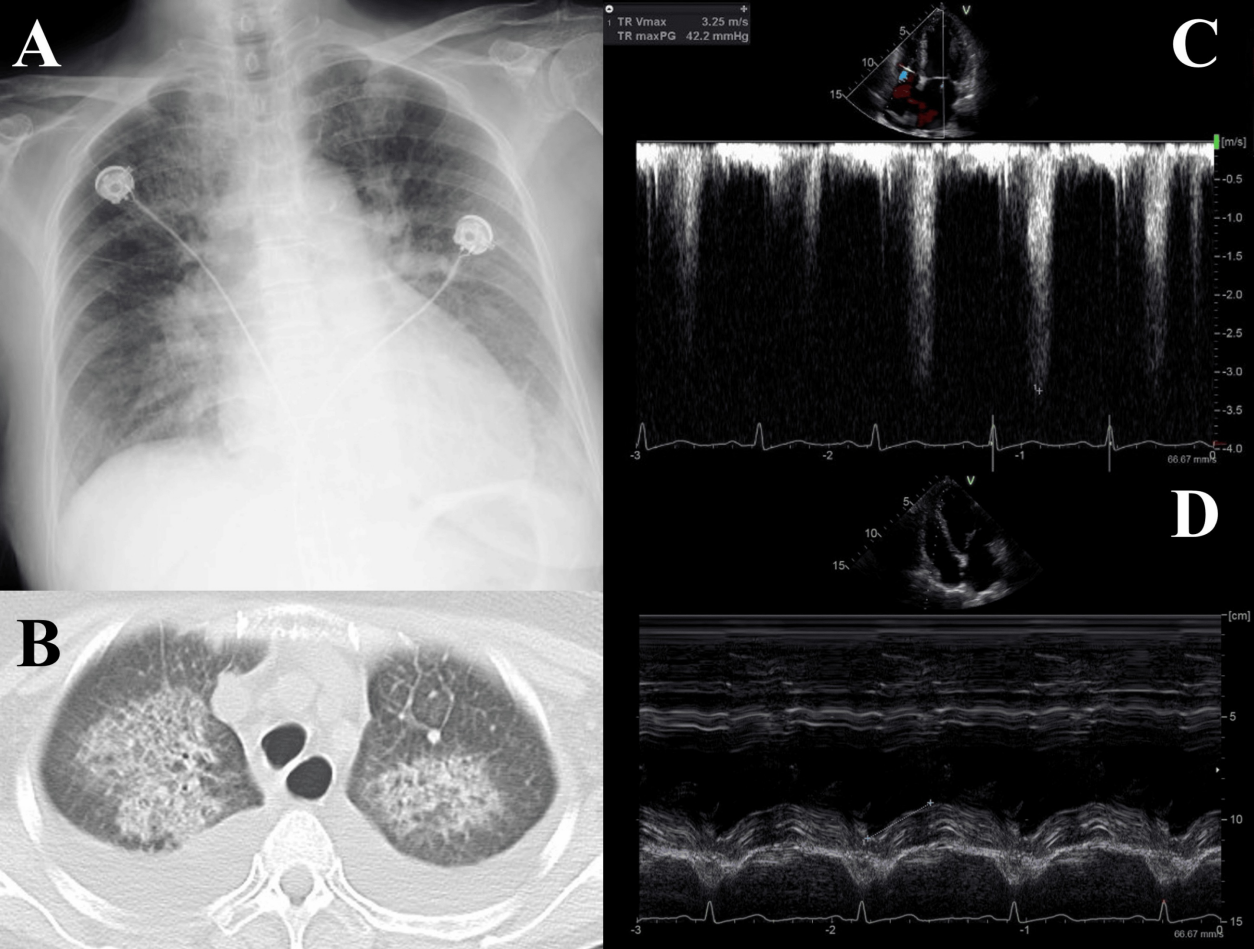
Image findings on hospital day 7, indicating cardiopulmonary involvement in transplant-associated thrombotic microangiopathy A. Chest X-ray showing cardiomegaly, bilateral pulmonary artery enlargement, and blunting of the costophrenic angles. B. Chest CT showing bilateral pulmonary opacities and pleural effusion. C. Continuous-wave Doppler tracing of tricuspid regurgitation showing peak velocity of 3.25 m/s, corresponding to estimated RVSP of 45 mmHg, suggesting pulmonary hypertension. D. Apical four-chamber view showing reduced TAPSE of 15.7 mm, suggesting right-sided heart failure. RVSP, right ventricular systolic pressure; TAPSE, tricuspid annular plane systolic excursion

From day 90, she gradually developed anemia, thrombocytopenia, and renal failure, but her vital signs remained within normal limits. Fluorine-18 fluorodeoxyglucose positron emission tomography/computed tomography (FDG-PET/CT) and bone marrow aspiration/biopsy showed no abnormalities. At an outpatient visit on day 130 (designated as hospital day 0), she presented with progressively worsening fatigue. Her vital signs were as follows: alert consciousness, body temperature 36.5°C, heart rate 91 bpm, blood pressure 154/82 mmHg, respiratory rate 18 /min, and SpO2 96 % on room air. Her baseline blood pressure during the HSCT hospitalization had been approximately 100/55 mmHg, indicating new-onset hypertension. Follow-up laboratory tests showed worsening values: hemoglobin 6.4 g/dL, platelets 12,000/µL, LDH 621 U/L, and creatinine 1.24 mg/dL. A peripheral blood smear revealed 1.2% schistocytes, raising suspicion for thrombotic microangiopathy (TMA). Accordingly, she was admitted for further evaluation and treatment. While undergoing evaluation for potential causes of TMA, she developed acute respiratory failure requiring >15 L/min of oxygen on hospital day 6. At this time, the NT-proBNP level was elevated to 48,666 pg/mL. Chest X-ray showed cardiomegaly, bilateral pulmonary artery enlargement, and blunting of the costophrenic angles (Figure 1A). Chest CT showed bilateral pulmonary opacities and pleural effusion (Figure 1B). Transthoracic echocardiography (TTE) demonstrated a continuous-wave Doppler tracing of tricuspid regurgitation with a peak velocity of 3.25 m/s, corresponding to an estimated right ventricular systolic pressure of 45 mmHg, suggesting PH (Figure 1C). Apical four-chamber view showed a reduced tricuspid annular plane systolic excursion of 15.7 mm, suggesting RHF (Figure 1D).

Noninvasive positive pressure ventilation (NPPV) was initiated in continuous positive airway pressure (CPAP) mode with a CPAP level of 5 cmH_2_O and an FiO_2_ of 0.5, which stabilized her cardiopulmonary status. Antihypertensive agents and furosemide were also administered. Given the presence of PH and RHF in TMA after HSCT, TA-TMA was strongly suspected. As shown in Tables 1, 2, the screening results were notable for normal coagulation, normal ADAMTS13 activity and inhibitor levels, negative direct and indirect Coombs tests, and the absence of paroxysmal nocturnal hemoglobinuria (PNH) clones. Additionally, stool tests were negative for enterohemorrhagic *Escherichia coli* (EHEC) and Shiga toxin. Based on these findings, disseminated intravascular coagulation (DIC), thrombotic thrombocytopenic purpura (TTP), autoimmune hemolytic anemia (AIHA), PNH, and hemolytic uremic syndrome (HUS) were excluded.

**Table 1:** Laboratory data Laboratory values relevant to the diagnosis of TA-TMA. Critical values are shown in bold. WBC, white blood cell; RBC, red blood cell; APTT, activated partial thromboplastin time; PT, prothrombin time; PT-INR, prothrombin time-international normalized ratio; FDP, fibrin degradation products; AST, aspartate aminotransferase; ALT, alanine aminotransferase; LDH, lactate dehydrogenase; ALP, alkaline phosphatase; ɤ-GTP, gamma-glutamyl transpeptidase; BUN, blood urea nitrogen; CCr, creatinine clearance; CRP, C-reactive protein

Laboratory Test (Unit)	Result	Reference Range
WBC (/µL)	2,600	3,100-8,400
Band (%)	0.5	1.0-7.0
Segmented (%)	57.5	34.0-70.0
Lymphocyte (%)	32.5	18.0-49.0
Monocyte (%)	7.5	2.0-10.0
Eosinophil (%)	1.5	0-8.0
Basophil (%)	0.5	0-2.0
RBC (×10^4^/µL)	194	380-480
Hemoglobin (g/dL)	6.4	12.0-16.0
Hematocrit (%)	19.5	34.0-45.0
Schistocyte (%)	1.2	<1.0
Reticulocyte (%)	7.1	0.2-2.5
Platelet (×10^4^/µL)	1.2	15-40
APTT (sec)	29.3	24-36
PT (sec)	12.6	10-14
PT-INR	0.95	0.9-1.1
Fibrinogen (mg/dL)	334	200-400
D-dimer (µg/mL)	0.9	<1.0
FDP (µg/mL)	3.0	<5.0
T-bilirubin (mg/dL)	3.1	0.2-1.2
D-bilirubin (mg/dL)	1.0	<0.4
AST (U/L)	18	13-30
ALT (U/L)	8	7-23
LDH (U/L)	621	124-222
ALP (U/L)	109	106-322
ɤ-GTP (U/L)	32	9-47
BUN (mg/dL)	17.8	8-20
Creatinine (mg/dL)	1.24	0.45-0.79
CCr (mL/min)	32.2	80-120
Total protein (g/dL)	5.1	6.5-8.0
Albumin (g/dL)	3.6	3.8-5.3
Uric acid (mg/dL)	8.5	3.7-7.0
Creatine kinase (U/L)	66	41-153
Na (mmol/L)	147	135-145
Cl (mmol/L)	110	98-110
K (mmol/L)	2.9	3.5-5.0
Ca (mg/dL)	8.0	8.4-10.6
P (mg/dL)	3.1	2.5-4.5
CRP (mg/dL)	1.22	<0.3
Haptoglobin (mg/dL)	<10	19-170
Urine occult blood	3+	Negative
Urine protein	2+	Negative
Urine WBC (/HPF)	1-4	<5
Urine RBC (/HPF)	5-9	<5
Urine hyaline cast (/LPF)	500	<2

**Table 2:** Laboratory data (continued) Laboratory values relevant to the exclusion of other diseases. Critical values are shown in bold. NT-proBNP, N-terminal pro-brain natriuretic peptide; NAP, neutrophil alkaline phosphatase; PNH, paroxysmal nocturnal hemoglobinuria; PAIgG, platelet-associated immunoglobulin G; Ab, antibody; CFH, complement factor H; RF, rheumatoid factor; ANA, anti-nuclear antibody; MPO-ANCA, myeloperoxidase anti-neutrophil cytoplasmic antibody; PR3, proteinase 3; GBM, glomerular basement membrane; Scl-70, topoisomerase I; RNP, ribonucleoprotein; dsDNS, double-stranded DNA; SS, Sjögren's syndrome; GPI, glycoprotein I; CL, cardiolipin; EBVCA, Epstein–Barr virus capsid antigen; EBEA, Epstein–Barr virus early antigen; HSV, herpes simplex virus; VZV, varicella-zoster virus; CMV, cytomegalovirus

Laboratory Test (Unit)	Result	Reference Range
NT-proBNP (pg/mL)	48,666	<125
KL-6 (U/mL)	140	<500
sIL-2R (U/mL)	558	121-613
Vitamin B12 (pg/mL)	1,169	200-914
Folate (ng/mL)	>25	>4.0
Direct Coombs	Negative	Negative
Indirect Coombs	Negative	Negative
Hemagglutination (titer)	32	<64
NAP score (points)	237	170-367
PNH cells	Negative	Negative
PAIgG (ng/10^7^Plt)	37	<46
Anti-platelet Ab	Negative	Negative
Anti-CFH Ab	Negative	Negative
C3 (mg/dL)	112	86-160
C4 (mg/dL)	31	17-45
CH 50 (U/mL)	50.4	20-45
RF (IU/mL)	<3	<15
ANA (titer)	<40	<40
MPO-ANCA (IU/mL)	0.2	<3.5
PR3-ANCA (IU/mL)	<0.6	<3.5
Anti-GBM Ab (U/mL)	<1.5	<3.0
Anti-Scl-70 Ab (U/mL)	<0.6	<10
Anti-centromere Ab (U/mL)	<0.4	<10
Anti-RNP Ab (U/mL)	0.7	<10
Anti-dsDNA Ab (IU/mL)	<0.6	<10
Anti-Smith Ab (U/mL)	<0.7	<10
Anti-SS-A Ab (U/mL)	<0.4	<10
Anti-SS-B Ab (U/mL)	<0.4	<10
Anti-Jo-1 Ab (U/mL)	<0.3	<10
Anti-β2GPI IgG (U/mL)	<0.7	<0.7
Anti-β2GPI IgM (U/mL)	0.6	<17.5
Anti-CL IgG (U/mL)	<4.0	<12.3
Anti-CL IgM (U/mL)	<2.5	<20.8
Lupus anticoagulant	1.1	<1.2
ADAMTS13 activity (IU/mL)	0.33	>0.1
ADAMTS13 inhibitor (BU/mL)	<0.5	<0.5
EBVCA IgG	3.3	<10
EBVCA IgM	0.3	<10
EBEA IgG	0.2	<10
EBV-DNA (copy/mL)	Negative	Negative
HSV IgG	4.5	<2.0
HSV IgM	0.17	<0.8
HSV-DNA (copy/mL)	Negative	Negative
VZV IgG	11.1	<2.0
VZV IgM	0.62	<0.8
VZV CF (titer)	<4	<4
CMV antigenemia	Negative	Negative
β-D-glucan (pg/mL)	<5.0	<20.0
Blood culture	Negative	Negative
Urine culture	Negative	Negative
Stool culture	Negative	Negative

The anti-complement factor H (CFH) antibody test was negative, and no variants were detected in complement regulatory genes such as *CFH*, *CFB*, *CFHR5*, *CFI*, *CD46*, *C3*, *THBD*, and *DGKE*. Accordingly, atypical HUS was excluded. In addition, all other microbiological and autoimmune screening tests were negative. Based on these results and the onset within 100 days post-HSCT, TA-TMA was diagnosed. The patient met the diagnostic criteria for TA-TMA established by the international expert panel in 2022, including the modified Jodele criteria [7] as well as those proposed by BMT-CTN [10], City of Hope [11], Jodele [12], and Cho [13]. Based on the risk stratification proposed in the same consensus, the patient was classified as high-risk TA-TMA [7].

**Figure 2:**
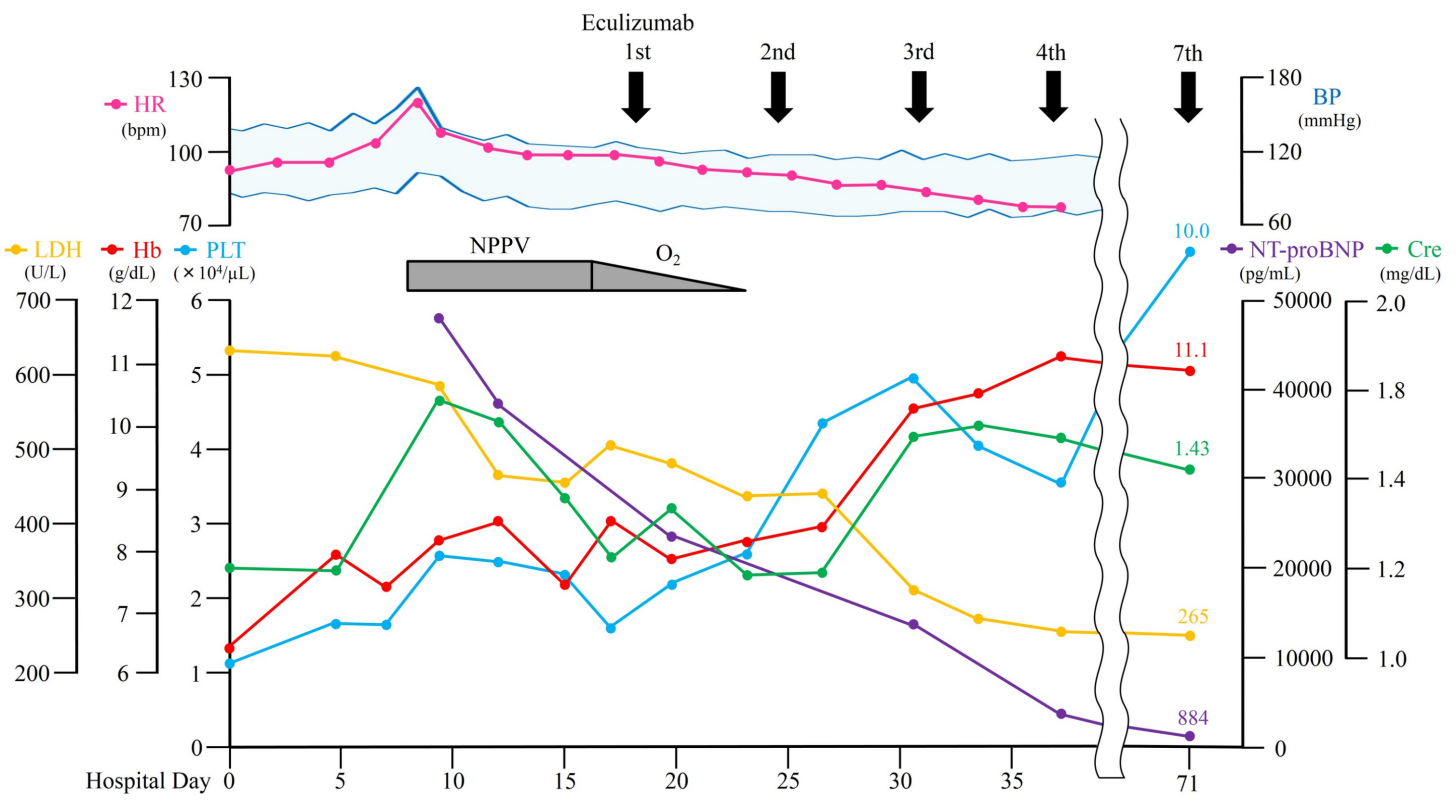
Clinical course showing the patient’s gradual improvement in vital signs, respiratory support requirements, and laboratory parameters over time HR, heart rate; BP, blood pressure; LDH, lactate dehydrogenase; Hb, hemoglobin; PLT, platelets; NPPV, noninvasive positive pressure ventilation; O2, oxygen; NT-proBNP, N-terminal pro-brain natriuretic peptide; Cre, creatinine

A quadrivalent meningococcal vaccine was administered on hospital day 8 prior to initiating eculizumab, a complement-targeting agent that increases the risk of meningococcal infection. Although the immune response may have been insufficient within two weeks of vaccination, earlier initiation of eculizumab was deemed preferable given the presence of severe complications, including PH and RHF. Consequently, eculizumab was administered on hospital day 17 at a dose of 900 mg weekly with concurrent antibiotic prophylaxis. Following treatment, malaise, dyspnea, tachycardia, and hypertension improved, accompanied by resolution of PH and RHF, allowing discontinuation of NPPV and supplemental oxygen. In addition, laboratory parameters, including hemoglobin, platelets, LDH, and NT-proBNP levels, gradually improved (Figure 2).

She was discharged on hospital day 39 and continues maintenance therapy with eculizumab 1,200 mg administered every two weeks. Clinical improvement has been sustained since then.

## Discussion

TA-TMA after autologous HSCT in adults is extremely rare. In particular, its occurrence following first-time autologous HSCT, as opposed to tandem autologous HSCT, is even rarer. Although autologous HSCT is widely used as a treatment for adults with malignant lymphoma and multiple myeloma, it is often performed by hematologists who may not have direct experience with allogeneic HSCT and, consequently, may be unfamiliar with TA-TMA, which is primarily recognized as a complication of allogeneic HSCT. When hemolytic anemia or thrombocytopenia is observed after autologous HSCT, such hematologists may not be able to diagnose TA-TMA, potentially leading to an unfortunate clinical outcome for the patient. Therefore, hematologists should consider TA-TMA in the differential diagnosis, not just cytomegalovirus infection or underlying bone marrow disorders [14]. It is essential to recognize that TA-TMA can occur not only after allogeneic HSCT or tandem autologous HSCT but also following first-time autologous HSCT in adults. Notably, being female or of advanced age, as exemplified by our patient, may represent additional risk factors that warrant heightened clinical awareness [3].

Next, TA-TMA has been widely reported as a disease with a poor prognosis [1,2]. In particular, it often leads to severe respiratory complications such as PH and diffuse alveolar hemorrhage, which are associated with a mortality rate exceeding 80% even with treatment using eculizumab [15,16]. However, these reports are mainly based on TA-TMA following allogeneic HSCT, and the prognosis is adversely affected independently by myeloablative conditioning regimens and GVHD. Although the diagnostic criteria for TA-TMA were established following consensus by the international expert panel in 2022 [7], the same criteria are used for both allogeneic and autologous HSCT despite their different levels of vascular endothelial damage, leading to ongoing ambiguity in clinical interpretation. Given that vascular endothelial damage plays a critical role in the pathogenesis of TA-TMA, establishing separate diagnostic criteria for TA-TMA after autologous and allogeneic HSCT may be necessary, as the latter involves more severe endothelial damage due to GVHD and myeloablative conditioning, which includes intensive chemotherapy and/or TBI. The prognosis of TA-TMA after autologous HSCT in adults is currently unknown, highlighting the need for additional research. Our patient showed clinical improvement with eculizumab even in the presence of serious complications such as PH and RHF, which suggests that the prognosis of TA-TMA after autologous HSCT is potentially more favorable than previously reported in allogeneic HSCT, with a lower mortality rate. Further studies are warranted to clarify the incidence rate, diagnostic criteria, and prognosis of TA-TMA after autologous HSCT.

## Conclusions

Although TA-TMA is primarily associated with allogeneic HSCT, this case highlights that it can also occur even after first-time autologous HSCT in adults despite its rarity. Early diagnosis and prompt initiation of complement-targeting agents, such as eculizumab, may lead to favorable outcomes even in cases complicated by life-threatening conditions such as PH and RHF. These findings suggest that complement inhibition may play a critical therapeutic role even in patients with cardiopulmonary complications and raise the possibility that the prognosis of TA-TMA after autologous HSCT may be more favorable than previously reported in allogeneic HSCT, with a lower mortality rate. Further accumulation of similar cases is needed to better understand the clinical course and prognosis of TA-TMA after autologous HSCT.
